# Non-Ultrasound-Guided Versus Ultrasound-Guided Femoral Venous Puncture in the Interventional Pulmonary Embolism Era: Does Ultrasound Guidance Improve Safety and Feasibility?

**DOI:** 10.31083/RCM46795

**Published:** 2026-09-16

**Authors:** Abdelrahman Elhakim, Desouki Abdelatti, Mohamed Elhakim, Osama Bisht, Ibrahim Yassin, Mohamed A Mosaad, Ahmad M Hassaan, Peter W. Radke, Mohammed Saad

**Affiliations:** ^1^Cardiology Department, Schoen Clinic Neustadt, 23730 Neustadt in Holstein, Germany; ^2^Pulmonology Department, University Wuppertal, 42119 Wuppertal, Germany; ^3^Intensive Care Medicine Department, The Royal Prince Alfred Hospital, Sydney, NSW 2050, Australia; ^4^Cardiology Department, Coswig Heart Center, 06869 Sachsen-Anhalt, Germany; ^5^Cardiology Department, Faculty of Medicine, Alazhar University, 11884 Cairo, Egypt; ^6^Cardiology Department, Sana Klinikum Coburg, 96450 Coburg, Germany

**Keywords:** pulmonary embolism, venous puncture, ultrasound, complications, femoral vein, catheter-based therapy

## Abstract

**Background:**

The use of interventional endovascular therapy for pulmonary embolism (PE) has become increasingly important over the last decade. In contrast to other cardiovascular interventions, these procedures carry a higher risk of access-related bleeding due to the acute clinical setting, elevated body mass index, and the frequent need for high-dose anticoagulation. However, there is still no clear consensus regarding the optimal vascular access site for endovascular PE therapy or the impact of ultrasound (US)-guided puncture on access-related complications.

**Methods:**

We treated 89 patients with intermediate- to high-risk PE using the Ekosonic endovascular system (EKOS). Group A (n = 39) underwent femoral venous puncture without US guidance, and Group B (n = 50) underwent US-guided femoral venous puncture.

**Results:**

Intraprocedural complications differed significantly between the two groups, with a higher incidence of vascular access complications in the non-US-guided group. Access-site pseudoaneurysms and retroperitoneal bleeding events occurred exclusively in the non-US-guided group (n = 6). Major (n = 3) and moderate (n = 5) bleeding events were also observed only in the non-US-guided group (*p* < 0.001). However, the total length of stay in the intermediate care unit and in the hospital did not differ between the groups. These findings are consistent with prior reports from other interventional settings that demonstrate fewer bleeding events among patients undergoing US-guided procedures.

**Conclusion:**

Widespread adoption of US-guided puncture in interventional PE therapy could reduce access-related complications, increase first-pass success, and reduce the number of puncture attempts, inadvertent arterial punctures, and unsuccessful access-site cannulations, particularly in patients with unfavorable access anatomy, obesity, or anatomical variations. Further education and implementation of US-guided access into training programs could improve the adoption of this technique.

## 1. Background

The role of interventional endovascular therapy for pulmonary embolism (PE) has increased substantially over the last decade [1,2,3,4]. In Europe, the femoral vein (FV) is the primary access route for small-bore interventional catheters in patients with PE.

Notably, patients who are undergoing interventional therapy for acute PE have an additional bleeding risk, as these patients are generally older, frail, and comorbid; therefore, these patients require concurrent anticoagulation therapy, which further increases the risk of access site-related bleeding. In addition, obesity is a well-recognized risk factor for PE. Obese patients with PE have a higher risk of extra puncture attempts, inadvertent arterial puncture, and unsuccessful cannulation.

Percutaneous vascular access can be achieved via traditional blind puncture guided by anatomical landmarks, ultrasound (US)-guided approaches, or fluoroscopy-guided techniques, all of which have gained widespread acceptance over the last decade. In addition, transfemoral access is a modifiable factor and carries a higher risk of complications than other venous access routes, such as transjugular or transbrachial access. Although access-related complications are the most frequently associated with PE endovascular therapy and can lead to prolonged in-hospital stays, increased blood transfusions, and higher in-hospital mortality, there is still no clear consensus on the optimal vascular access site, nor on whether US-guided puncture reduces access-related complications.

In contrast, several studies in the fields of transcatheter aortic valve implantation (TAVI) and percutaneous coronary interventions (PCIs) have demonstrated that US-guided puncture may be associated with improved safety and reduced access-related adverse outcomes.

Historically, the femoral artery (FA) was the preferred vascular access site for PCI. However, the increasing recognition of access-related complications has prompted the development and adoption of strategies to improve this technique.

In recent years, randomized controlled trials (RCTs) have demonstrated that radial artery access is superior to FA access with respect to access-site complications and survival. Therefore, recent guidelines recommend radial access as the default technique for PCI, which has led to a marked increase in radial access procedures [5]. Nonetheless, FA access remains the gold-standard approach for structural and complex high-risk percutaneous interventions.

The impact of US-guided FA access on access-site complications has been extensively investigated in PCI and in large-bore arterial access procedures for structural heart interventions. Despite some conflicting evidence regarding the role of US-guided puncture, several meta-analyses of RCTs have demonstrated that US guidance is associated with a reduction in bleeding events.

Meanwhile, the potential benefits of US-guided FV puncture in the era of interventional PE therapy have not yet been studied. Strategies to reduce access site-related complications may lower morbidity and mortality and streamline discharge pathways, thereby reducing postoperative length of stay and improving safety, effectiveness, and economic outcomes.

With the growing number of interventional PE therapies being performed, preventive and prompt management strategies to reduce vascular access-related complications are essential for decreasing the clinical burden of PE and improving outcomes. Therefore, this prospective study aimed to compare traditional blind puncture versus US-guided puncture in intermediate- to high-risk PE patients treated with the EkoSonic Endovascular System (EKOS) (Boston Scientific, Marlborough, MA, USA) as a catheter-based therapy.

## 2. Methods

This was a retrospective, non-randomized, single-center cohort study designed and conducted at Schoen Clinic Neustadt in Holstein, Germany. Approval was obtained from the institutional review board at the University of Lübeck (reference number 21-172), and written informed consent was obtained from all patients.

The sample size determination and statistical analyses were performed by the Institute of Medical Biometry and Statistics at the University of Lübeck, Germany.

All intermediate- and high-risk PE patients who did not improve under anticoagulation therapy were evaluated for treatment with the interventional EKOS system.

Several studies using the EKOS system have demonstrated favorable safety outcomes compared with systemic fibrinolysis and improved hemodynamics compared with anticoagulation alone [6]. The system has previously received approval from the United States Food and Drug Administration (FDA) for the treatment of acute intermediate- and high-risk PE [1,6,7,8,9].

Interventional PE therapy with EKOS in intermediate- to high-risk PE patients was initiated in August 2020, using traditional blind punctures. However, a trend toward vascular-access-related complications with subsequent prolonged hospital stays prompted us to reconsider this approach. Consequently, we changed our practice and began performing US-guided punctures in June 2023.

### 2.1 Sample Size Calculation

According to the ULTraound Accelerated ThrombolysIs of PulMonAry Embolism (ULTIMA) study [7], the smallest relevant difference requires 39 analyzable patients per group (https://rpact-cloud.share.connect.posit.cloud (Sereetz, Germany; accessed on 6 August 2025)). The sample size was calculated for a one-sample *t*-test (one-sided): H0mu = 0; H1: effect = 3.2; standard deviation = 7.8; significance level = 5%; power = 80%.

### 2.2 Anticoagulation

Before the procedure, intravenous unfractionated heparin was initiated with a target activated partial thromboplastin time (PTT) of 60–80 seconds, and the PTT was monitored every 1–2 hours. After completion of EKOS therapy, intravenous unfractionated heparin was discontinued, and all patients received oral anticoagulation. The drug, dose, frequency, and duration of anticoagulation were selected by the attending physician according to the comorbidities of each patient.

### 2.3 Study Population

Patients who met the following criteria were eligible for inclusion:

- High-risk of PE (simplified pulmonary embolism severity index (sPESI) >1, right ventricle/left ventricle (RV/LV) ratio >0.9, and positive cardiac biomarkers) with systemic arterial hypotension >20 mmHg, cardiogenic shock, or resuscitation.

- An intermediate- to high-risk of PE (sPESI >1, RV/LV ratio >0.9, and positive cardiac markers) with deterioration of symptoms or hemodynamics (prolonged systemic arterial hypotension >20 minutes, cardiogenic shock, respiratory rate >20/min, oxygen saturation <90%, and heart rate >100/min).

Exclusion criteria were as follows:

- Active intracranial or intraspinal bleeding within 6 months, or ischemic stroke within 3 months.

- Active bleeding from a major organ, or major surgery with a high bleeding risk within 7 days.

### 2.4 US-Facilitated and Catheter-Directed Low-Dose Fibrinolysis

The FV was the primary vascular access throughout the study. After catheter placement, baseline right and left pulmonary artery pressures were measured via the catheters, and patients were transferred to the intermediate care unit to initiate EKOS therapy. No adjunctive interventional techniques to assist thrombus removal or dissolution were permitted.

A fixed-dose regimen of tissue plasminogen activator (t-PA) was used: 6 mg at 1 mg/h for 6 h with saline coolant at 35 mL/h for unilateral PE (one drug delivery device was placed), or 12 mg at 1 mg/h for 6 h with saline coolant at 35 mL/h for bilateral PE (two drug delivery devices were placed).

A total of 89 patients with intermediate- to high-risk PE who underwent interventional PE therapy with the EKOS system were included. The study population was divided into two subgroups: group A (EKOS arm with blind puncture, n = 39) and group B (EKOS arm with US-guided puncture, n = 50). A study flowchart is shown in Fig. 1.

**Fig. 1.**
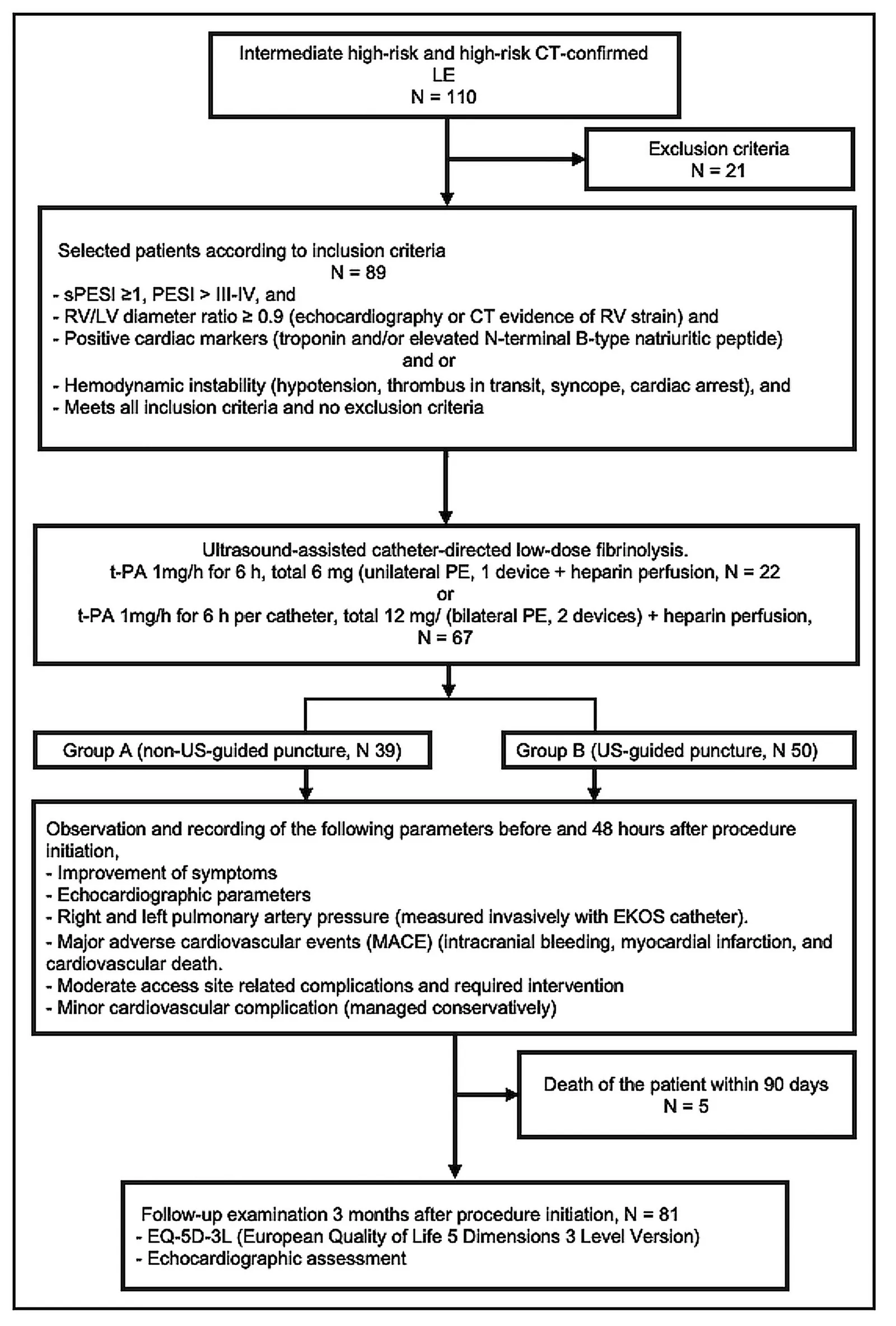
**Study flowchart**. A total of 110 patients with acute intermediate- to high-risk pulmonary embolism (PE) were screened for eligibility, of whom 89 were eligible for enrolment. Regarding pulmonary artery pressure measurements: 6 h for invasive methods and 48 h for non-invasive methods. CT, computed tomography; SPESI, simplified pulmonary embolism severity index; RV/LV, right ventricle/left ventricle; US, ultrasound; EKOS, EkoSonic Endovascular System.

### 2.5 Protocol for US-Guided Puncture

One or two puncture sites were selected according to the underlying PE pathology (unilateral or bilateral disease).

### 2.6 General Technical Adjustments

The general technical adjustments included lowering the probe frequency for deeper penetration, placing the entry point 1–3 cm distal to the probe, using a shallower needle angle (30–40°), and selecting a longer needle if required.

#### 2.6.1 Pre-Procedural Assessment and Access Strategy

The common FV was identified as the primary access site using a high-frequency linear vascular US probe (5–12 MHz). A systematic US survey was performed before puncture to:

Confirm vein patency, exclude venous thrombosis, differentiate vein from artery (based on compressibility and Doppler characteristics), identify anatomical variations, determine vessel depth and diameter, and assess relationship to the common femoral artery. This pre-precedural analysis aimed to avoid unintended arterial puncture and unsuccessful cannulation.

Both short-axis (transverse) and long-axis (longitudinal) views were routinely evaluated before needle insertion. After local US-guided anesthesia was performed, US-guided needle advancement was performed using both “out-of-plane” and “in-plane” techniques, with the needle oriented at approximately 45° to the skin until adequate venous tenting was observed at 12 o’clock in the short-axis view at the intended puncture site [10].

#### 2.6.2 Definitions of Imaging Planes

Short-axis view (out-of-plane technique): The probe was positioned perpendicular to the vessel so that the vessel appeared circular on the screen; the needle was typically advanced out of plane, which is the optimal view for identifying the artery–vein relationship.

Long-axis view (in-plane technique): The probe was positioned parallel to the vessel course so that the vessel appeared tubular on the screen; the needle was advanced in-plane, allowing continuous visualization of the shaft and tip, which is optimal for tracking the needle (Table 1).

**Table 1.** **Comparison of short-axis and long-axis modes of view**.

Mode of view	Short-axis/out-of-plane	Long-axis/in-plane
Advantages	Better visualization of the vein in relation to the artery helps to avoid accidental arterial puncture. Shorter learning curve	Needle course and tip can be visualized, which reduces the risk of penetrating the posterior vessel wall
Disadvantages	Needle is only visualized as an echogenic point	Longer learning curve

#### 2.6.3 Standardized Mode Selection Criteria

A. Recommended strategy for patients with normal body mass index (BMI) (18.5–25 kg/m^2^):

Initial vessel identification was performed in the short-axis view, followed by puncture using either short-axis + out-of-plane (standardized 45° entry) or long-axis + in-plane (for experienced operators).

Rationale: Vessel depth was usually 1–2 cm, with clear vessel borders and low soft-tissue attenuation; both modes provide reliable visualization.

B. Recommended strategy for overweight/obese patients (BMI >30 kg/m^2^):

A pre-scan was performed in the short-axis view, followed by puncture preferably conducted in the long-axis view with an in-plane technique.

Rationale: Increased vessel depth (>2–3 cm) makes needle visualization more difficult in the out-of-plane mode; the long-axis view allows continuous needle tip tracking and reduces the risk of posterior wall puncture.

In the event of a puncture failure, the needle and wire were removed. After applying manual pressure to the vein for about 5 min, a second puncture was attempted. Once venous access was achieved, the needle was removed, and a 6 Fr sheath was inserted over a 0.035-inch guidewire. The 0.035-inch wire was then advanced, and US was used to confirm the appropriate intravascular course. If resistance was encountered during advancement, the wire was repositioned under fluoroscopy until it reached the iliac vein and then the inferior vena cava.

### 2.7 Statistical Analysis

Qualitative variables are presented as counts and proportions, and quantitative variables as the mean and standard deviation. Categorical variables are presented as numbers (n) and percentages (%). Qualitative variables were compared between the treatment groups using the chi-square test or Fisher’s exact test, as indicated. The distribution of quantitative variables was assessed for normality using the Shapiro–Wilk test. As the data were normally distributed, parametric tests were used. Between-group comparisons were conducted using an unpaired *t*-test. Within-group comparisons of pulmonary artery outcomes between two time points were evaluated using a paired *t*-test. For echocardiography outcomes measured at more than two time-points, repeated-measures analysis of variance (ANOVA) was used. In the case of a significant difference, pairwise comparisons between time points were performed, with the *p*-value adjusted for multiple comparisons using the Bonferroni correction. Correlations between the quantitative variables were analyzed using Pearson’s correlation coefficient.

All tests were two-sided, and a *p*-value < 5% was considered statistically significant. Statistical analyses were performed using the IBM SPSS version 27 software package (IBM Corp., Armonk, NY, USA).

### 2.8 Safety Outcomes

Safety outcomes included vascular complications related to the access site, bleeding events, and all-cause in-hospital mortality. Bleeding events were not classified according to any established bleeding score, in line with the latest ESC atrial fibrillation guideline [11].

Therefore, bleeding events were classified as either minor bleeding managed conservatively, moderate bleeding requiring intervention (e.g., drainage or thrombin injection), or major, life-threatening bleeding requiring advanced life support and intervention.

### 2.9 Efficacy Measures

Efficacy was assessed by comparing baseline values with post-treatment measurements, including:

- Reduction in invasive pulmonary artery pressures.

- Improvement in echocardiographic parameters (RV function, RV/LV ratio, systolic pulmonary artery pressure (SPAP)).

- Assessments were performed at 48 hours and at 90-day follow-up.

## 3. Results

### 3.1 Baseline Characteristics

The baseline characteristics of the study population are listed in Table 2.

**Table 2.** **Baseline demographics and clinical characteristics**.

	Non-US (n = 39)	US (n = 50)	*p*-value
Age, year	67.77 ± 13.19	67.0 ± 13.92	0.792
Height, cm	173.31 ± 9.63	173.48 ± 10.28	0.936
Weight, kg	89.62 ± 21.53	90.28 ± 23.6	0.891
Body mass index, kg/m^2^	29.72 ± 6.79	29.88 ± 9.88	0.929
Female	20 (51.3%)	27 (54%)	0.799
Ethnicity/race	White	White	
Comorbid conditions	38 (97.44%)	43 (86%)	0.061
Hypertension	15 (38.46%)	15 (30%)	0.402
Diabetes mellitus	1 (2.56%)	1 (2%)	0.859
Smoking	1 (2.56%)	4 (8%)	0.269
Obesity	32 (82.05%)	29 (58%)	0.015
Immobility	6 (15.38%)	15 (30%)	0.107
Operation	3 (7.69%)	12 (24%)	0.041
Previous DVT	2 (5.13%)	8 (16%)	0.107
Previous PE	1 (2.56%)	7 (14%)	0.061
Atherosclerotic cardiovascular disease	1 (2.56%)	2 (4%)	0.709
Anemia, g/dL	15 (38.46%)	32 (64%)	0.016
Stroke	5 (12.82%)	1 (2%)	0.043
Active cancer	6 (15.38%)	6 (12%)	0.643
Chronic pulmonary disease	6 (15.38%)	15 (30%)	0.107
Renal insufficiency (GFR <90 mL/min)	5 (12.82%)	26 (52%)	<0.001
Hyperlipidemia	3 (7.69%)	1 (2%)	0.198
Rheumatic disease	4 (10.26%)	0 (0%)	0.020
Estrogen use	1 (2.56%)	0 (0%)	0.255
Congenital blood disease	3 (7.69%)	0 (0%)	0.046

Values are mean ± SD, standard deviation or n (%). PE, pulmonary embolism; DVT, deep vein thrombosis; GFR, glomerular filtration rate.

The mean patient age was 67 years. Common risk factors for PE included obesity (68%); a mean BMI of 29 kg/m^2^, anemia (53%), hypertension (30%), an active cancer (26%), chronic renal disease (68%), and rheumatic disease (9%).

### 3.2 Baseline Characteristics of PE

All patients had symptomatic PE confirmed by a contrast-enhanced computed tomography (CT) image of the thorax (Table 3). The duration of PE symptoms was ≤14 days. Intermediate- to high-risk and high-risk PE were observed in 71 (80%) and 18 (20%) patients, respectively.

**Table 3.** **Characteristics of PE**.

Symptoms		Non-US (n = 39)	US (n = 50)	*p*-value
Dyspnea III (symptoms with minimal exertion)		26 (66.7%)	21 (42%)	0.021
Dyspnea IV (symptoms at rest)		2 (5.1%)	19 (38%)	<0.001
	Cardiopulmonary resuscitation	3 (7.7%)	0 (0%)	0.046
	Syncope	7 (17.9%)	3 (6%)	0.077
	Palpitation	0 (0%)	2 (4%)	0.206
	Dizziness (vertigo)	1 (2.6%)	5 (10%)	0.165
PE risk stratification				
	High risk	3 (8%)	8 (16%)	0.237
	Intermediate high risk	36 (92%)	42 (84%)	
Diagnosed DVT				
	Right	10 (25.6%)	14 (28%)	0.803
	Left	8 (20.5%)	7 (14%)	0.415
Oxygen saturation, %		89.89 ± 5.9	87.92 ± 9.06	0.244
Breathing frequency, minute		21.16 ± 4.51	21.08 ± 4.66	0.938
Heart rate, minutes before EKOS therapy		94.82 ± 13.48	102.5 ± 18.05	0.029
Heart rate, minute after EKOS therapy		81.38 ± 7.07	78.68 ± 9.34	0.136
Blood pressure, mmHg				
	Systole	140.08 ± 23.23	135.08 ± 29.84	0.391
	Diastole	83.72 ± 15.18	82.44 ± 20.45	0.745
Computed tomography–PE				
	Right	2 (5.1%)	2 (4%)	0.722
	Left	1 (2.6%)	3 (6%)	
	Bilateral	36 (92.3%)	45 (90%)	
ECG				
	Sinus rhythm	20 (51.3%)	17 (34%)	0.057
	Sinus tachycardia	14 (35.9%)	29 (58%)	
	Atrial flutter	5 (12.8%)	2 (4%)	
	Atrial fibrillation	0 (0%)	2 (4%)	
Laboratory parameters				
Troponin, ng/mL		523.79 ± 702.27	391.94 ± 472.08	0.322
NT-Pro-BNP, pg/mL				
	Before therapy	5044.33 ± 8707.52	3920.76 ± 5004.57	0.475
	After therapy	4089.06 ± 7122.78	3310.27 ± 3645.15	0.505
Lactate, mmol/L				
	Before therapy	2.67 ± 2.27	2.12 ± 0.84	0.156
	After therapy	2.04 ± 3.02	1.28 ± 0.37	0.124
Hemoglobin, mg/dL				
	Before therapy	12.84 ± 2.24	13.01 ± 1.99	0.704
	After therapy	11.09 ± 2.10	11.61 ± 1.46	0.197
CRP, mg/dL		37.11 ± 35.39	48.91 ± 39.62	0.148
Creatinine, mg/dL		1.11 ± 0.37	1.11 ± 0.39	0.998
GFR, mL/min		65.53 ± 25.05	64.28 ± 25.18	0.815
TSH, mLU/L		1.62 ± 0.74	1.75 ± 1.42	0.617

Values are mean ± SD or n (%). ECG, electrocardiogram; NT-Pro-BNP, N-terminal pro-B-type natriuretic peptide; CRP, C-reactive protein; TSH, Thyroid-Stimulating Hormone.

### 3.3 Procedural Characteristics

The right femoral vein was the primary route for device placement (88%). US guidance was used in 56% of the vascular access procedures for catheter placement. Bilateral devices were placed through a double-access approach in 75% of patients (Table 4).

**Table 4.** **Procedural characteristics**.

		Non-US (n = 39)	US (n = 50)	*p*-value
Successful device placement		100%	100%	1.0
No of devices per patient				
	1	4 (10.3%)	18 (36%)	
	2	35 (89.7%)	32 (64%)	
Access site				
	Right femoral vein	36 (92%)	42 (84%)	0.348
	Left femoral vein	3 (8%)	4 (8%)	
	Right jugular vein	0 (0%)	2 (4%)	
	Left jugular vein	0 (0%)	2 (4%)	
Completed infusion of t-PA		37 (95%)	49 (98%)	0.417
Duration of investigation/minute		10.57 ± 6.62	10.06 ± 9.82	0.783
Radiation dosage in cGY/cm^2^		1645.19 ± 877.25	911.50 ± 946.30	<0.001

Values are mean ± SD or n (%). t-PA, tissue-type plasminogen activator; cGY/cm^2^, centigray per square centimeter; SD, standard deviation; n, number.

The mean total dose of t-PA was 6 mg for unilateral PE or 12 mg for bilateral PE (Table 4). All 89 devices (100%) were successfully placed. A total of three patients in the non-US-guided puncture arm did not undergo follow-up invasive pulmonary artery monitoring after the 6-hour window or echocardiography measurements performed within the 48-hour window (Tables 5,6).

**Table 5.** **Primary pulmonary artery pressure efficacy outcomes**.

PA/mmHg	Baseline	6 h	Mean difference (95% CI)	*p*-value
Right systole pressure	47.11 ± 14.86	27.86 ± 10.71	19.25 (15.79–22.71)	<0.001
Right diastole pressure	20.44 ± 9.14	14.33 ± 7.63	6.11 (3.84–8.39)	<0.001
Right mean pressure	30.02 ± 8.83	19.17 ± 7.67	10.85 (8.70–13.01)	<0.001
Left systole pressure	45.27 ± 14.49	28.88 ± 9.85	16.29 (12.96–19.83)	<0.001
Left diastole pressure	20.62 ± 7.04	15.39 ± 6.82	5.24 (3.32–7.15)	<0.001
Left mean pressure	29.90 ± 8.75	20.63 ± 7.43	9.27 (7.05–11.49)	<0.001
Total mean systolic pressure	46.19 ± 13.29	28.37 ± 9.21	17.82 (14.68–20.69)	<0.001

CI, confidence interval; PA, pulmonary artery. Patients with complete pulmonary artery measurements = 89.

**Table 6.** **Echocardiography efficacy outcomes**.

Echocardiography	Baseline	48 hours	90 days	*p*-value	Baseline–48 h	Baseline–90 days	48 h–90 days
RVEDD, mm	45.35 ± 5.07	37.28 ± 4.62	32.43 ± 3.52	<0.001	8.07 (6.75–9.39); *p* < 0.001	12.92 (11.33–14.52); *p* < 0.001	4.86 (3.63–6.08); *p* < 0.001
LVEDD, mm	36.86 ± 6.44	41.71 ± 5.09	43.29 ± 4.17	<0.001	–4.84 (–6.29 to –3.39); *p* < 0.001	–6.44 (–7.97 to –4.90); *p* < 0.001	–1.59 (–2.49 to –0.69); *p* < 0.001
RV/LV ratio	1.22 ± 0.27	0.91 ± 0.16	0.76 ± 0.11	<0.001	0.31 (0.23–0.39); *p* < 0.001	0.46 (0.39–0.53); *p* < 0.001	0.15 (0.11–0.19); *p* < 0.001
TAPSE, mm	17.15 ± 4.89	21.73 ± 3.30	22.56 ± 2.48	<0.001	–4.58 (–5.75 to –3.41); *p* < 0.001	–5.41 (–6.84 to –3.98); *p* < 0.001	–0.83 (–1.80–0.15); *p* = 0.126
SPAP, mmHg	51.23 ± 9.28	33.54 ± 9.51	27.46 ± 7.69	<0.001	17.69 (15.13–20.24); *p* < 0.001	23.77 (21.10–26.44); *p* < 0.001	6.09 (3.65–8.52); *p* < 0.001
TAPSE/SPAP, mm/mmHg	0.35 ± 0.14	0.69 ± 0.22	0.88 ± 0.24	<0.001	–0.35 (–0.40 to –0.29); *p* < 0.001	–0.53 (–0.59 to –0.46); *p* < 0.001	–0.18 (–0.24 to –0.12); *p* < 0.001

RVEDD, right ventricular end-diastolic diameter; LVEDD, left ventricular end-diastolic diameter; SPAP, systolic pulmonary artery pressure; RV/LV, right ventricle-to-left ventricle ratio; TAPSE, tricuspid annular plane systolic excursion. Echocardiographic measurements were complete for 89 patients. The *p*-value < 0.001, after adjustment for multiple comparisons using the Bonferroni correction.

One patient with high-risk PE died of retroperitoneal bleeding and septic shock 4 h after the initiation of therapy. Determining the exact cause of death was challenging, given the acute clinical condition and the potential contribution of bleeding related to low-dose fibrinolysis, anticoagulation therapy, or both. A second patient experienced retroperitoneal bleeding and shock. Surgical pulmonary thrombectomy and hematoma evacuation were successfully performed.

The third patient with a high-risk PE died at the initiation of therapy and had a baseline hemoglobin level of 6 mg/dL, severe comorbidities, and a short period of pulseless electrical activity.

Four moderate bleeding events occurred after the intervention, without any lasting complications (Figs. 2,3; Tables 7 and 8).

**Fig. 2.**
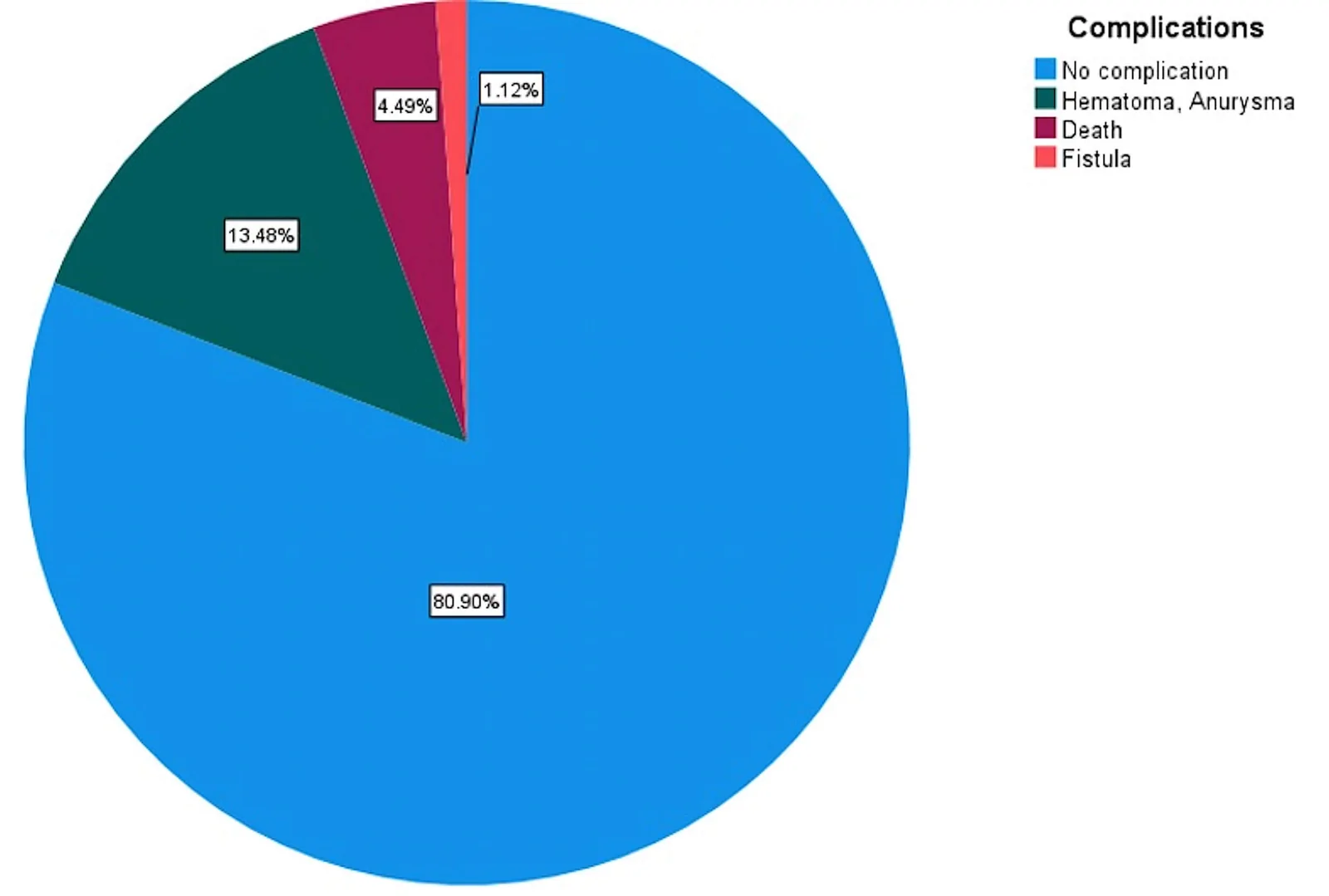
**Overall safety outcomes during hospital stay (n = 89)**. There were three pseudoaneurysms (one managed with thrombin injection), five moderate or large hematomas, three bleeding events (in the lung, knee, and rectal mucosa) that were all successfully drained, and three life-threatening bleeding events (one retroperitoneal bleed managed surgically).

**Fig. 3.**
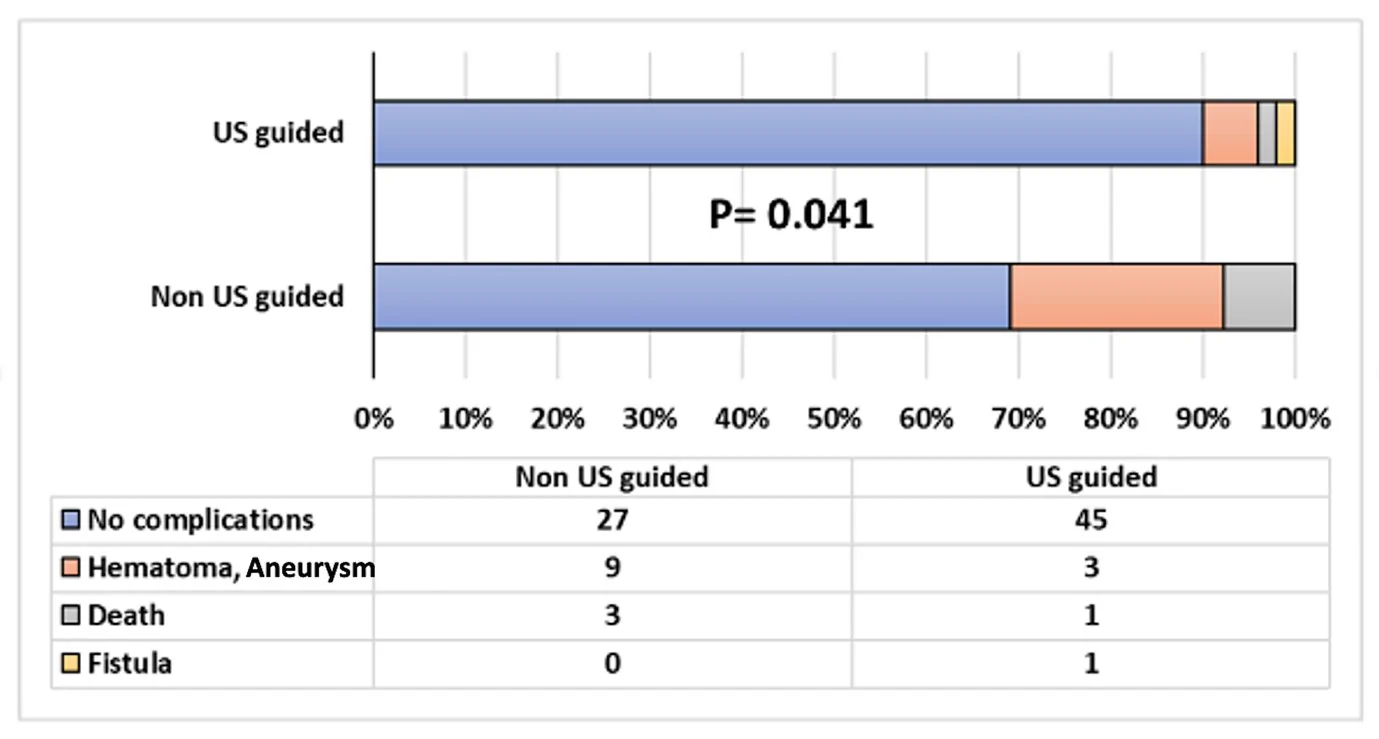
**Subgroup analysis during hospital stay with a significant reduction in access site complications in the US-guided group (n = 50) versus the non-US-guided group (n = 39)**. US, ultrasound.

**Table 7.** **Safety outcomes**.

		Non-US	US	*p*-value
Length of stay/days		6.87 ± 4.04 (SD 4.035)	5.67 ± 1.94 (SD 1.940)	0.138
Complications		12 (30.8%)	5 (10%)	0.013
ICU stay		1.16 ± 0.49 (SD 0.495)	1.11 ± 0.54 (SD 0.538)	0.701
Serious and severe adverse events potentially related to device		0 (0%)	0 (0%)	-
Serious and severe adverse events potentially related to t-PA		3 (7.7.%)	0 (0%)	<0.001
Major bleeding within 90 days				
	Moderate (required intervention)	5 (13%)	0 (0%)	<0.001
	Severe (life threatening)	3 (6.8%)	0 (0%)	
EQ-5D-3L		0.95 ± 0.07	0.98 ± 0.05	0.053
Patient reported subjective clinical improvement		77.34 ± 12.31	81.48 ± 16.26	0.268

EQ-5D-3L, European quality–five dimension–three level; ICU, intensive care unit; PA, pulmonary artery; US, ultrasound. Quantitative variables are presented as mean ± SD, and qualitative variables are presented as frequency (percentage).

**Table 8.** **Summary of moderate and major bleeds of group A vs. No major or moderate events were observed in group B**.

Bleeding event	Site of bleed	Transfused blood products	Hgb (mg/dL) Baseline–24 h	Outcome and complications
Access site hematoma	5 (5.6)		11.6–11.3	Recovered
			12.8–10.1	Recovered
			18.7–15.2	Recovered
			17.6–14.1	Recovered
			10.7–7.7	Recovered
Access site pseudoaneurysm	3 (3.4)		14–11.4	Thrombin injection Recovered
			13.4–12.5	Recovered
			15–13	Recovered
Access site bleeding	3 (3.4) Retroperitoneal	2 U PRBCs 4 U PRBCs -	15.5– 6.3 14.4–7.5 15–10	Died of PE, comorbidity Surgically evacuated Conservative
Mucosal bleeding	1 (1.1), unclear	2 U PRBCs	7.5–6.8	Died of PE and comorbidity
	1 (1.1) rectal hematoma		12.1–10.8	Rectal drainage, recovered
Left hemothorax	1 (1.1) 2nd day after lung resection, under heparin	1 U PRBCs	11–8.7	Thorax drainage, recovered
Left knee	1 (1.1) left knee		14–11.4	Knee joint drainage, recovered
Anemia	7 (7.9)	1–4 U PRBCs case by case		Recovered

Hgb, hemoglobin; PRBC, packed red blood cell; PE, pulmonary embolism.

Five patients with septic shock, recurrent ischemic stroke, and colon cancer died within 90 days. Pulmonary artery pressure was measured invasively 6 hours after the initiation of therapy (Table 5; Fig. 4). We observed a decrease in the mean right and left pulmonary artery pressures from 30 and 46 mmHg at baseline to 19 and 28 mmHg, respectively, corresponding to mean differences of 10 and 17 mmHg (*p* > 0.001; Fig. 4).

**Fig. 4.**
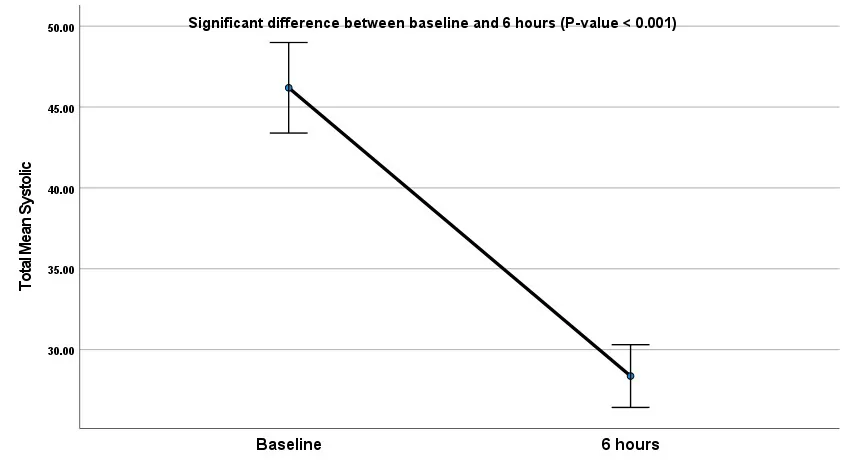
**Invasive measurements of pulmonary artery pressure from baseline to 6 h after therapy showed a significant reduction in all treated patients**.

Recovery of the RV systolic function, assessed using the tricuspid annular plane systolic excursion/systolic pulmonary artery pressure (TAPSE)/(SPAP) ratio, increased from 0.35 mm/mmHg at baseline to 0.69 within 48 h and to 0.88 at the 90-day follow-up (Fig. 5).

**Fig. 5.**
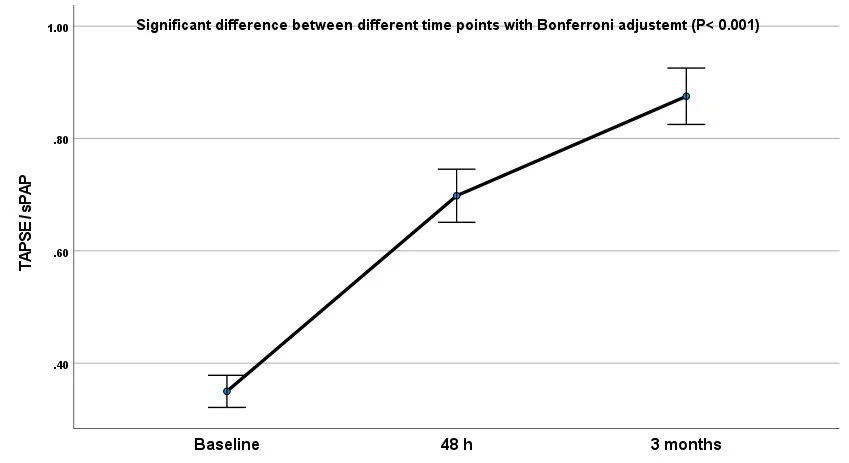
**Echocardiographic TAPSE/SPAP ratio measured at baseline, 48 h, and 90 days after therapy initiation showed significant improvement over time**. TAPSE, tricuspid annular plane systolic excursion; SPAP, systolic pulmonary artery pressure.

The mean RV/LV pressure ratio decreased from 1.2 at baseline to 0.91 within 48 h, and to 0.76 at the 90-day follow-up, with a mean difference of 0.46 (*p* < 0.001; Table 6; Figs. 6, 7). This study demonstrates a significant, but weak correlation between the RV/LV ratio measured by echocardiography and by CT (Fig. 6), and between invasive and noninvasive systolic pulmonary artery pressure measured by right heart catheterization and echocardiography before initiation of treatment (Fig. 7; Table 9).

**Fig. 6.**
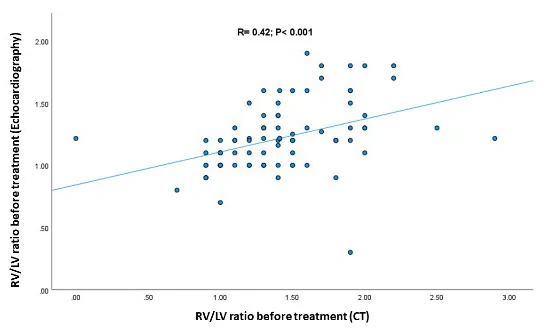
**A weak but significant correlation between the RV/LV ratio measured by echocardiography and computed tomography before initiation of treatment**. RV/LV, right ventricular/left ventricular ratio.

**Fig. 7.**
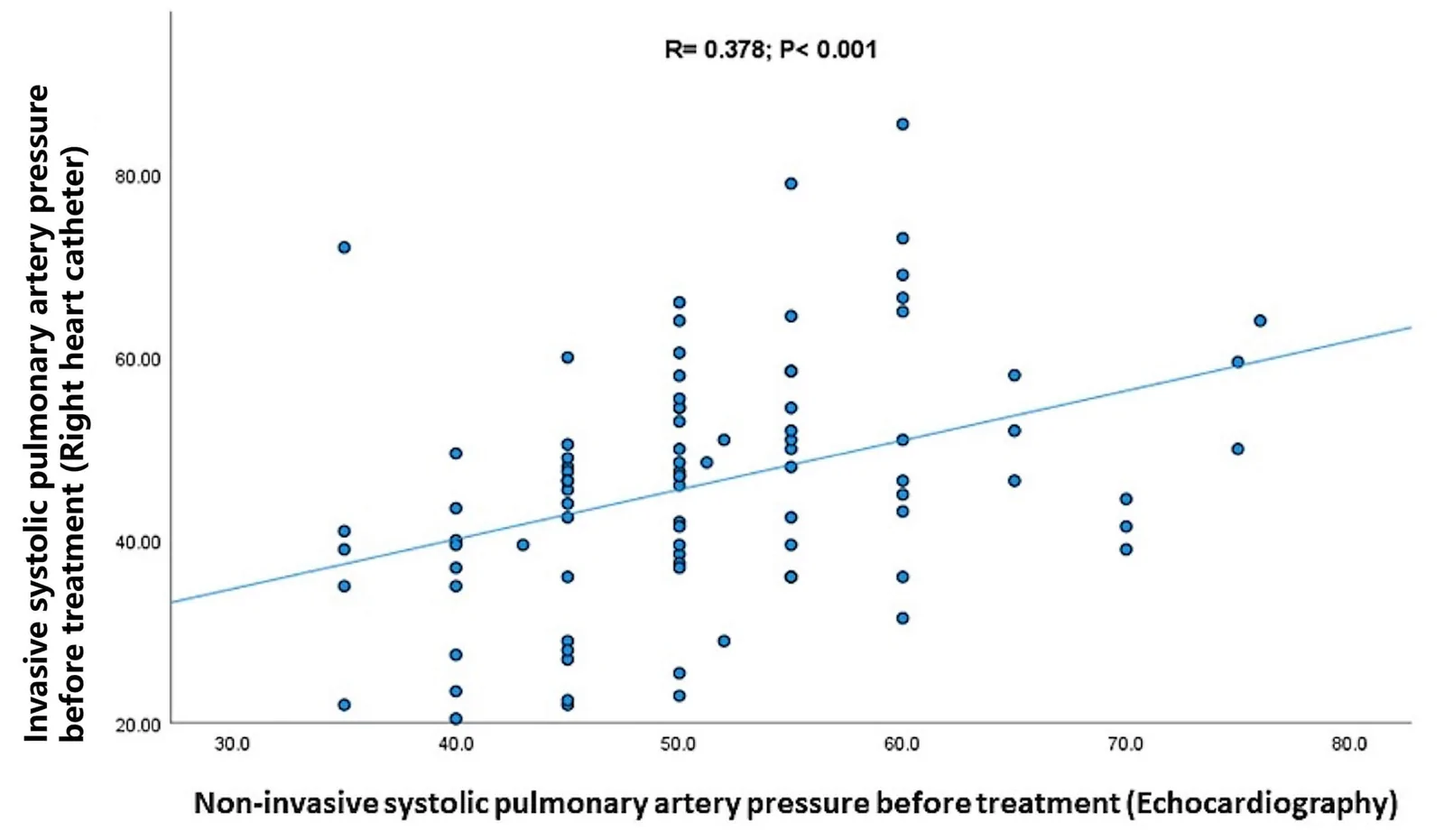
**A weak but significant correlation between invasive and noninvasive systolic pulmonary artery pressure measured by right heart catheterization and echocardiography respectively before initiation of treatment**.

**Table 9.** **Correlation between computed tomography and echocardiographic parameters**.

Correlation (n = 89)	Pearson correlation coefficient	*p*-value
RV/LV ratio (Echo)–RV/LV ratio (CT)	0.42	<0.001
RVEDD (Echo)–RVEDD (CT)	0.15	0.158
LVEDD (Echo)–LVEDD (CT)	0.59	<0.001
SPAP -Total invasive systolic	0.378	<0.001

CT, computed tomography; RV, right ventricle; LV, left ventricle; RVEDD, right ventricular end-diastolic diameter; LVEDD, left ventricular end-diastolic diameter; SPAP, systolic pulmonary artery pressure; Echo, echocardiography.

The mean improvement in symptoms was 96% on the European Quality of Life 5 Dimensions 3 Level Version (EQ-5D-3L) quality-of-life questionnaire (0 = the worst health you can imagine; 100 = the best health you can imagine) at 90-day follow-up.

## 4. Discussion

This prospective study demonstrated that in patients with intermediate- to high-risk PE treated with EKOS therapy, US-guided puncture of the FV was associated with significantly better safety outcomes and fewer major bleeding events than non-US-guided puncture.

A significantly higher first-attempt success rate was observed in the US-guided group, whereas multiple puncture attempts and accidental arterial puncture were observed in the non-US-guided group (n = 13). Access-site-related complications occurred in 12 (30%) patients in the non-US-guided group and five (10%) in the US-guided group (*p* = 0.013). Major (n = 3) and moderate (n = 5) bleeding events occurred exclusively in the non-US-guided group (*p* < 0.001). These included three pseudoaneurysms (one treated with thrombin injection), five hematomas, three bleeding events in the lung, knee, and rectal mucosa, which were drained successfully, and three life-threatening bleeding events, which included one retroperitoneal bleed, were managed surgically.

Total bleeding events (life-threatening, major, moderate, and minor) were also increased in the non-US-guided group (*p* = 0.05). We found a significantly higher risk of bleeding events (odds ratio (OR) 0.13, *p* = 0.0007) and combined access-related events (OR 0.45, *p* = 0.0069) in the non-US-guided group. In addition, we conducted a propensity score analysis using baseline patient characteristics (sex, hypertension, diabetes mellitus, peripheral vascular disease, dyspnea class III or IV, BMI, and age) to control for potential confounding factors. The adjusted OR for bleeding events was 0.13 (*p* = 0.002), and the adjusted OR for combined access-related complications was 0.44 (*p* = 0.01).

Radiation exposure was higher in the non-US-guided group (1645.19 ± 877.25 centigray per square centimeter (cGY/cm^2^)), likely due to the early phase of the learning curve and limited operator experience.

Our findings are consistent with the broader interventional literature, which shows fewer bleeding events and improved safety profiles with US-guided vascular access. However, the total length of intermediate care unit and overall hospital stay did not differ significantly between the two groups, although there was a slight increase in hospital length of stay in the non-US-guided group: 6.87 ± 4.04 (group A) vs. 5.67 ± 1.94 days (group B); *p* = 0.138. This difference may be explained by the higher complication rate observed in the non–US-guided group.

Regarding obese patients (i.e., those with a BMI >25 kg/m^2^), both groups were obese with a BMI of 29 kg/m^2^.

However, we observed a clinical reduction in both bleeding and vascular access-related complications in the US-guided group. Therefore, this study confirms that US-guided puncture has potential benefits in the overweight subsets with unfavorable access anatomy.

Overall, strategies to optimize PE therapy and management should be developed to reduce cardiovascular burden and improve outcomes. Vascular access complications have a major impact on prognosis and mortality, as these complications are associated with a prolonged hospital stay, secondary infection, need for blood transfusion, and increased mortality.

Risk factors for vascular access complications include high BMI, use of large-bore sheaths, high or low puncture site, combined arterial and venous puncture, current anticoagulation medication, and failed manual compression. Vascular-access-related complications during or after interventions include vessel dissection, bleeding, hematoma, fistula, and the formation of pseudoaneurysms. A steady decline in these complications can be attributed to increasing operator experience, use of low-profile sheaths, US-guided puncture, and advances in vascular closure techniques.

In addition, PE patients with high BMI (≥30 kg/m^2^) require high-dose anticoagulation, as obesity significantly affects drug distribution, dosing strategies, and the risk–benefit balance. Anti-Xa monitoring may be necessary in patients with BMI ≥40, weight >150 kg, and/or renal dysfunction. Apixaban and rivaroxaban are generally effective in obese patients; however, evidence remains limited in cases of extreme obesity (BMI >50) [12,13,14,15]. Preventive strategies should be implemented through peri-interventional risk assessment, including clinical evaluation, US, and radiological preprocedural workup. In addition, widespread adoption of US-guided puncture, alternative access sites, and further miniaturization of sheath diameters may be modifiable risk factors that could help further reduce access-related PE and endovascular therapy-related complications.

However, if a complication arises, streamlined pathways and standardized algorithms are essential to optimize management. Hematoma is the most common access-site complication. Anticoagulation increases the risk of hematoma even when manual compression is adequate. In most cases, hematomas can be treated conservatively with additional manual compression. Surgical evacuation is indicated when a large hematoma compresses nerves or adjacent structures.

In addition, pseudoaneurysm can occur after vessel rupture with continuous extravascular arterial bleeding into a pseudo-capsule due to failure of the vascular closure device, improper manual compression, especially in obese patients, concurrent use of anticoagulation, and underlying peripheral vessel disease. On clinical examination, a pseudoaneurysm is most commonly diagnosed as a pulsatile mass in the groin, often accompanied by a systolic bruit on auscultation and Doppler US.

Small pseudoaneurysms (<2.5 cm) can be treated conservatively with US-guided compression for 20–40 minutes, followed by band compression for the next 8–24 hours, which can lead to thrombosis of the pseudoaneurysm. Larger pseudoaneurysms can cause progressive discomfort, local infection, septic embolism, compression of the surrounding structures, and rupture. In these cases, US-guided interventional thrombin injection at incremental doses of 0.2–0.4 mL can be continued until the flow within the pseudoaneurysm ceases. Meanwhile, covered stent implantation or surgical repair with suturing or patch plasty may also be required [16,17,18].

Moreover, the most life-threatening complication related to the access site is retroperitoneal hemorrhage or hematoma, which can result from injury to the femoral, inferior epigastric, iliac, renal, or aortic vessels. A hematoma typically presents with nonspecific symptoms, such as groin, flank, or back pain. Early diagnosis and identification of the bleeding source are determinants of management outcomes. Bedside US is the initial diagnostic setup, followed by abdominal CT to confirm the underlying pathology and identify the bleeding source. Most cases can be managed conservatively with transfusion of red blood cell units if indicated. In cases of active bleeding, endovascular coil embolization of small vessels and covered stent-graft implantation, or even surgical evacuation and vessel repair, may be required, depending on the size and location of the vessel rupture and hemodynamic status [19].

Furthermore, access-site infections are more often reported after surgical cut-down. Therefore, attempts to manage vascular-access-related complications should first be addressed with endovascular interventions. Superficial infections typically respond well to local and/or systemic therapy, whereas surgical debridement and vacuum-assisted closure (VAC) therapy (Kinetic Concepts; KCI Medical, San Antonio, TX, USA) may be required for deep wound infections and those with prolonged healing.

Although gaps remain in the existing evidence for interventional PE therapy and there is a lack of consensus guidelines on the use of US-guided puncture, this study supports the routine adoption of US-guided venous puncture, as this approach leads to improved safety outcomes, fewer access-related complications, earlier mobilization, and shorter hospital stays.

Most cardiovascular interventional studies have shown a potential benefit of US-guided femoral venous puncture, with improvements in technical outcomes, including fewer puncture attempts, higher first-pass success, and fewer accidental arterial punctures.

In addition, US guidance can facilitate first-pass success, reduce extra puncture attempts, minimize inadvertent arterial punctures and unsuccessful cannulations, and account for anatomical orientation and variations. An electrophysiological study of catheter ablation of arrhythmias (n = 254) classified the relationship between the femoral artery and FV into four types: Type I (vein parallel to artery), Type II (vein medial to artery with ≤50% overlap), Type III (vein posterior to artery with >50% overlap), and Type IV (vein lateral to artery). At the lower inguinal level, Type II accounted for 70.7%, while Types III and IV accounted for 23.4% and 5.9%, respectively. Conversely, most cases (92.5%) exhibited Type II, followed by Type I (6.5%), in the upper inguinal region [20].

Moreover, central venous catheter (CVC) placement has traditionally been performed using anatomical landmark techniques, which do not account for anatomical variations, venous thrombosis, or occlusion.

Therefore, a joint guideline from the Society of Cardiovascular Anesthesiologists and the American Society of Echocardiography recommended the use of real-time US for CVC placement in the internal jugular vein (IJV) (category A, level 1 evidence). However, the available evidence remains insufficient to support routine US use for the FV, and current recommendations have not been revised (category C, level 2 evidence) [21]. Thus, additional studies are required to evaluate the impact of US-guided FV puncture on reducing access-site-related complications.

A meta-analysis that compared anatomic landmark techniques with US-guidance for CVC placement in the IJV with regard to the peri-interventional complications demonstrated that the use of US for CVC placement in the IJV reduced the overall complication rate compared with conventional landmark techniques (US, 48 complications in 1212 patients (4.0%) vs. landmark, 161/1194 (13.5%); risk ratio (95% confidence interval (CI)) 0.29 (0.17–0.52)) [22].

Another study of 72 patients compared the safety and effectiveness of US-guided subclavian vein puncture with the traditional blind puncture technique. The puncture time was significantly reduced in the traditional group, while the puncture success rate was higher in the US-guided group [23].

A meta-analysis of seven RCTs including 3180 patients undergoing invasive procedures concluded that US-guided access, compared with the conventional technique, was associated with a significant reduction in access-site hematoma (1.2% vs. 3.3%), vascular complications (1.3% vs. 3.0%), and a non-significant 0.7% absolute risk reduction in major bleeding events [24].

Another meta-analysis of eight observational TAVI studies (n = 3875) compared access-site vascular complications following transfemoral access using US versus conventional fluoroscopy. A US-guided approach was significantly associated with a reduced risk of total access-site vascular complications (odds ratio 0.50 (95% CI 0.35–0.73)) [25].

In electrophysiology, an RCT in patients undergoing catheter ablation for atrial fibrillation (AF) on uninterrupted anticoagulation therapy (n = 320) randomized patients 1:1 to US-guided versus conventional venipuncture. Interestingly, the intra-procedural outcome measures favored the US approach (inadvertent arterial puncture 0.07 vs. 0.25; *p* < 0.001; extra puncture attempts 0.5 vs. 2.1; *p* < 0.001; unsuccessful cannulation 0.6% vs. 14%; *p* < 0.001; first pass success, 74% vs. 20%; *p* < 0.001; puncture time, 288 vs. 369 s; *p* < 0.001). However, the complication rates did not differ between the two arms (0.6% vs. 1.9%; *p* = 0.62) [26].

The main reasons for the limited adoption of US-guided venous puncture among interventional cardiologists include the unavailability of US devices in the cardiac catheterization laboratory, a steep learning curve, and the belief that the US technique is more time-consuming. Further education and implementation within interventional cardiology training programs could improve adoption of the technique.

## 5. Limitations

The main limitations of this study are its single-center, non-randomized design, a relatively small sample size, and the lack of long-term follow-up data of up to one year.

However, the overall sample size was sufficient to yield statistically meaningful results. In addition, baseline characteristics between the groups were imbalanced. Patients in the non‑US‑guided group were more often obese than those in the US‑guided group (72% vs. 64%) and included more high‑risk PE patients who required cardiopulmonary resuscitation (n = 3 (7%)). Conversely, US‑guided patients had more comorbidities, including anemia, renal dysfunction, active pulmonary conditions, immobility, previous PE, and/or deep vein thrombosis.

Moreover, vascular access complications can be markedly influenced by the learning curve, operator experience, and familiarity with US-guided puncture. Furthermore, the patient groups were naturally divided by duration, which may introduce a time-dependent bias. However, the same operators performed the interventions in both groups, potentially introducing learning-curve bias. Therefore, these findings should be considered hypothesis-generating. The results must be interpreted with caution, as the study design does not allow for causal inferences.

Hence, further, larger randomized studies are needed to analyze the impact of US-guided percutaneous puncture in reducing access-related complications, to understand the most favorable access site for endovascular PE therapy, such as transfemoral, transbrachial, and transjugular, and to potentially inform future guideline recommendations supporting adoption of this technique as the gold standard in clinical practice.

## 6. Conclusion

Vascular access complications have a major impact on outcomes and mortality in patients with PE. Compared with other cardiovascular interventions, interventional PE procedures carry an additional access-related bleeding risk due to the acute setting, high BMI, and the need for high doses of anticoagulant therapy.

Strategies to reduce access site-related complications can lower morbidity and mortality, streamline discharge pathways, and may potentially reduce postoperative length of stay and overall PE burden, thereby improving both safety and economic outcomes.

Widespread adoption of US-guided puncture may improve outcomes by reducing access-related interventional PE complications, favoring first-pass success, and decreasing extra puncture attempts, inadvertent arterial punctures, unsuccessful cannulation, and reliance on unfavorable access anatomy in obese patients and in patients with anatomical variation. Larger multicenter randomized studies are needed to confirm these findings and the benefits of US-guided venous cannulation in patients with PE.

## Data Availability

All data were archived safely in a safe dataset during this study. All data are available on request taking into consideration the polices of patient privacy.

## Abbreviations

AF, Atrial fibrillation; BMI, Body mass index; cGY/cm^2^, centigray/square centimeter; CT, Computed tomography; CVC, Central venous catheter; ECG, Electrocardiogram; EKOS, EkoSonic Endovascular System; EQ-5D-3L, European quality–five dimension–three level; IJV, Internal jagular vein; FV, Femoral vein; LV, Left ventricle; LVEDD, Left ventricle end diastolic diameter; PE, Pulmonary embolism; PTT, Partial thromboplastin time; RBBB, Right bundle branch block; RCT, Randomized controlled trial; RV, Right ventricle; RVEDD, Right ventricle end diastolic diameter; SPAP, Systolic pulmonary artery pressure; TAPSE, Tricuspid annular plane systolic excursion; TAVI, Transcatheter Aortic Valve Implantation; T-PA, Tissue plasminogen activator; US, Ultrasound.
